# Micronucleus frequency in children exposed to biomass burning in the Brazilian Legal Amazon region: a control case study

**DOI:** 10.1186/1472-6831-12-6

**Published:** 2012-03-08

**Authors:** Herbert Ary Sisenando, Silvia Regina Batistuzzo de Medeiros, Paulo Artaxo, Paulo HN Saldiva, Sandra de Souza Hacon

**Affiliations:** 1 Escola Nacional de Saúde Pública - ENSP, Fiocruz, Rio de Janeiro CEP: 21041-210, RJ, Brazil; 2 Departamento de Biologia Celular e Genética, UFRN, Natal CEP: 59072-970, RN, Brazil; 3 Departamento de Física Aplicada, USP São Paulo CEP: 05508-900, SP, Brazil; 4 Departamento de Patologia, USP, São Paulo CEP: 01246-903, SP, Brazil; 5 Departamento de Patologia, UFF, Niterói CEP: 24033-900, RJ, Brazil

## Abstract

**Background:**

The Amazon represents an area of 61% of Brazilian territory and is undergoing major changes resulting from disorderly economic development, especially the advance of agribusiness. Composition of the atmosphere is controlled by several natural and anthropogenic processes, and emission from biomass burning is one with the major impact on human health. The aim of this study was to evaluate genotoxic potential of air pollutants generated by biomass burning through micronucleus assay in exfoliated buccal cells of schoolchildren in the Brazilian Amazon region.

**Methods:**

The study was conducted during the dry seasons in two regions of the Brazilian Amazon. The assay was carried out on buccal epithelial cells of 574 schoolchildren between 6-16 years old.

**Results:**

The results show a significant difference between micronucleus frequencies in children exposed to biomass burning compared to those in a control area.

**Conclusions:**

The present study demonstrated that in situ biomonitoring using a sensitive and low cost assay (buccal micronucleus assay) may be an important tool for monitoring air quality in remote regions. It is difficult to attribute the increase in micronuclei frequency observed in our study to any specific toxic element integrated in the particulate matters. However, the contribution of the present study lies in the evidence that increased exposure to fine particulate matter generates an increased micronuclei frequency in oral epithelial cells of schoolchildren.

## Background

The Amazon extends from the Atlantic Ocean to the eastern slopes of the Andes, with 61% of the area belonging to Brazil, where it is called the Brazilian Amazon. The region has been environmentally affected by advancing economic development, especially agribusiness and ranching. This has provoked changes in land use, resulting in increased deforestation and increased biomass burning in both native forest and pasture areas [1,2]. Several studies show that pasture formation is the main land use in newly deforested regions [3]. Sugar cane is an example of agribusiness that is in rapid expansion in the Amazon biome, driven by the political incentive to produce and consume biofuel both nationally and internationally. Brazil is one of the largest producers of biofuel worldwide, with most production concentrated in the Midwest region. Sugarcane bagasse burning at harvest time is widely practiced in Brazilian production to facilitate harvesting and increase the yield of manual cutting; however, this archaic procedure results in increased pollutant concentrations in the atmosphere [4-7].

Biomass burning is a process of combustion of organic matter that is characterized by a multielemental composition and size distribution [8,9], representing a combined exposure to several pollutants and health effect associated with multiple exposure. PM has the ability to adsorb many mutagenic compounds present in the environment and transport them through the respiratory branches to the alveolar capillary basement membrane [10]. Fine and ultra fine particles are specially toxic to cells [11]. The presence of a complex mixture of air pollutants may be able, at high concentrations or after long-term exposure, to induce genotoxic damage in human cells [12]. The main genotoxic damage generated by exposure to pollutants from biomass burning are oxidative types of damage (e.g.: 8-oxodG) and formation of DNA adducts, which if not repaired may cause mutation in DNA [13-15].

Among the bioassays used to evaluate the impact of environmental, genetic and life-style factors on genomic stability in humans, the micronucleus assay (MN-assay) has been highlighted for its easy use, and by requiring lower capital investment associated with precision obtained by scoring larger numbers of cells [16]. Use of oral epithelial cells as a bioindicator of genotoxic damage arising from exposure to environmental carcinogens via inhalation and the digestive tract can be explained by the ability of cells to metabolize carcinogens and for being the first physical barrier of these tracts, resulting in greater incidence of human cancers in epithelial cells [17]. Micronuclei (MN) are structures resulting from whole chromosomes or chromosomal fragments that lag behind at anaphase during nuclear division, allowing us to detect the action of clastogenic and aneugenic agents [17-19].

Children are more susceptible to the effects of exposure to air pollution, especially respiratory diseases in terms of harm to human health associated with exposure to biomass burning pollutants [20-23]. A study conducted by Huen et al. [24] in children and adults through oral micronucleus assay suggested greater vulnerability to traffic-related air pollution in children. This sensitivity of children to air pollution can be explained by a higher level of physical activity and spending more time outside and therefore they are more exposed to outdoor air pollution [25], as well as immaturity of the lung and immune systems [26]. Biomonitoring studies in children are affected to a lower extent by confounders like cigarette smoking and alcoholic drinking habits, occupational exposure and life-style (e.g.: diet type), which are factors of great concern in adults [16]. The relationship between exposure to air pollutants and formation of micronuclei in buccal cells of children was described by Huen et al. [24] in the United State of America, Iurchenko et al. [27] and Maimulov et al. [28] in Russia, Sycheva et al. [29] in Vietnam and Lahiri et al. [30] in India.

The aim of this study was to assess genotoxicity potential of biomass burning pollutants through micronucleus assay in exfoliated buccal cells of schoolchildren in the Brazilian Amazon region.

## Methods

### Study site

The study was conducted in two test areas (Tangará da Serra (TS) and Porto Velho (PV)) and one control area (Chapada dos Guimarães (CH)). Tangará da Serra (TS) has a population of 83,431 inhabitants distributed over an area of 11,391.314 km^2^, located in a transition area between the Amazon and Cerrado biomes and has the characteristics of an urban area within Amazon region; the region has the largest sugarcane production and contains the two largest factories in the southern Brazilian Amazon [31-33]. Porto Velho (PV) has a population of 428,527 inhabitants distributed over an area of 34,096.429 km^2^, located in a rural district on the banks of the Madeira River in the Amazon biome; the population has characteristics typical of Amazonian riverbank dwellers [33]. The two test areas are in a region with typical cycles of drought and rain that alter air pollution levels, and lies in the dispersion path of the pollution plume resulting from burnings in the Legal Amazon and pollution from neighboring countries, thus impacting respiratory health of the population exposed, especially morbidity in schoolchildren [6,34,35].

Chapada dos Guimarães (CH) has a population of 17,821 inhabitants distributed over an area of 5,983.595 km^2 ^and, was selected as the control area due its better air quality. There is no industrial production or sugar cane burning and automobile traffic is light, compared to the other municipalities involved. It was also chosen for the similarities in meteorological variables (rainfall, temperature and humidity) compared to other municipalities involved in this study [6,32,33].

**Figure 1:**
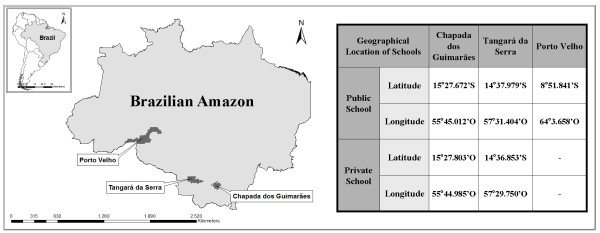
**Geographical location of sites of study, delimiting the region of the Brazilian Amazon**.

Latitude and longitude coordinates of all points collected in the study areas are shown in Figure 1.

### Micronucleus assay in exfoliated buccal cells

The MN-assay was carried out on the buccal epithelial cells of 574 children between 6-16 years old (446 children directly exposed to burning of biomass and 128 children living in the control area). Buccal cell samples were collected during the burning (dry) season in the region, which occurs between the months of May and October. Samples from TS and CH were collected in August 2008, and samples from PV were collected in August 2009. The samples were obtained by rubbing the inside of the cheeks of study subjects with a cytobrush. Cells were collected in sample bottles containing 5 mL of buffer solution (0.1 M EDTA, 0.01 Tris-HCl and 0.02 M NaCl, pH 7) and transported to the laboratory. Samples were processed according to the protocol described by Heuser et al. [36], and slides were evaluated according to criteria established by Tolbert et al. [37]. A total of 2,000 cells/child were analyzed using an optical microscopy at a magnification of 1000 X.

In order to reduce confounding factors, the study excluded smokers, alcoholics, patients or individuals with any genetic malformations. The influence of economic power as a confounding factor was assessed with collection of buccal cells in public and private schools when possible. Other factors such as age and gender were also evaluated. All slides were analyzed in a coded fashion by the same person.

The study was approved by the Ethics Committee of the National School of Public Health (ENSP)/Oswaldo Cruz Foundation (Fiocruz) under CAAE number 0054.0.031.000-08. All parents/guardians of the children were informed about the purpose of the study, as well as they signed a document of participation and consent with study.

### Environmental data collection

The daily means of fine particulate matter (PM_2.5_) and of meteorological variables (humidity and temperature) in the region under the direct influence of burnings and in the control area, during the dry season, were obtained from CATT-BRAMS (Coupled Aerosol and Tracer Transport model of the Brazilian Regional Atmospheric Modeling System) accessed through the Environmental Information Integrated to Environmental Health System (http://sisam.cptec.inpe.br/msaude/). A more detailed description of this model was presented by Freitas et al. [38].

### Statistical analysis

The data of micronuclei frequency (MN‰) were evaluated by variance analysis (ANOVA), followed by Tukey's comparison test. The data of PM_2.5 _were evaluated by Mann-Whitney's test. Significance was determined when compared with data obtained in control area. All data were analyzed using SPSS 16.0.

## Results

Figure 1 shows the Legal Amazon region and municipalities involved in this study, in addition to the georeferencing of all the points sampled in the oral micronucleus assay.

Table 1 shows the mean concentration of environmental (PM_2.5_) and meteorological (temperature and humidity) variables modeled by CATT-BRAMS in all municipalities studied. In relation to meteorological variables, we observed that temperature and humidity profiles were very similar in the areas evaluated. Point PV showed higher humidity content (68 and 72%) in both periods modeled (2008 and 2009) compared to the other points (CH and TS). With regard to the environmental variable, we found that PM_2.5 _presented a profile in which modeled concentrations in the test areas were significantly higher (TS = 21 and 22 μg/m^3^; PV = 19 and 16 μg/m^3^) than those found in the control area (10 and 12 μg/m^3^), during both periods.

**Table 1:** Distribution of environmental and meteorological data of the study sites in the years 2008 and 2009.

Environmental data	Temperature (°C)		Humidity (%)		PM_2.5 _(μg/m^3^)	
	
	2008	2009	2008	2009	2008	2009
**Chapada dos Guimarães (CH)**	26	25	51	62	10	12

**Tangará da Serra (TS)**	25	24	57	68	21^##^	22^##^

**Porto Velho (PV)**	28	27	68	72	19^##^	16^##^

Data obtained in the dry season (May-October). ^##^*p *< 0.01 by Mann-Whitney test.

The results presented in Table 2 show a statistically significant difference between MN‰ obtained in the test areas (TS = 1.43 ± 0.84 and PV = 1.13 ± 0.63) compared with the control area (CH = 0.29 ± 0.41). Results from TS and PV showed an accumulation of micronucleus frequency of 4.9 and 3.8 times higher than that observed in the control area, respectively. The study also assessed the influence of other parameters such as school type, gender and age (years) on MN‰. The results show that micronuclei frequency obtained in schoolchildren exposed to biomass burning was significantly higher (TS = Private school: 1.30 ± 0.4 and Public school: 1.46 ± 0.9; PV = Public school: 1.13 ± 0.63) than that observed in the control area (CH = Private school: 0.27 ± 0.36 and Public school: 0.3 ± 0.44). Regarding school type, the results showed no significant difference (*p > 0.05*) between results from public schools in relation to private schools. When comparing MN‰ obtained in public schools in TS and PV, one sees that micronucleus frequency of students from TS (1.46 ± 0.9) was slightly higher than in PV (1.13 ± 0.63), however not significantly so. Regarding gender, there was no significant difference between MN‰ of male and female students in all areas studied. No difference regarding the age parameter was observed when comparing ages at each site. In general, significant differences were observed when comparing data obtained in sites TS and PV compared to CH in all parameters, except between MN‰ in children under 07 years old from PV that was not significant in comparison to the control (*p > 0.05*).

**Table 2:** Micronucleus frequency of exfoliated buccal cells in children.

Micronucleus Frequency (MN‰)	Chapada dos Guimarães (CH)			Tangará da Serra (TS)			Porto Velho (PV)		
	**N**	**Mean**	**SD**	**N**	**Mean**	**SD**	**N**	**Mean**	**SD**

**By site**	128	0.29	± 0.41	245	1.43 **	± 0.84	201	1.13 **	± 0.63

Others									

parameters:									

**Type of school**									

Private school	57	0.27	± 0.36	42	1.30 **	± 0.40	-	-	-

Public school	71	0.30	± 0.44	203	1.46 **	± 0.90	201	1.13 **	± 0.63

**Gender**									

Male	54	0.27	± 0.36	110	1.50 **	± 0.82	108	1.08 **	± 0.61

Female	74	0.30	± 0.44	135	1.39 **	± 0.85	93	1.18 **	± 0.65

**Age (years)**									

≤ 7	18	0.19	± 0.31	46	1.20 **	± 0.83	14	0.89	± 0.56

8-9	28	0.29	± 0.46	55	1.33 **	± 0.87	27	1.06 *	± 0.54

10-11	28	0.27	± 0.40	59	1.57 **	± 0.83	32	1.11 **	± 0.74

12-13	27	0.31	± 0.40	57	1.54 **	± 0.92	49	1.10 **	± 0.69

≥ 14	27	0.35	± 0.43	28	1.54 **	± 0.51	79	1.22 **	± 0.58

*p < 0.05 and **p < 0.01 by Tukey's test. N = Number of children examined. SD = Standard Deviation.

## Discussion

Among the few studies assessing genetic damage in schoolchildren exposed to air pollution, none has assessed damage generated by exposure to pollutants released through biomass burning in the Amazon region.

A study conducted by Sisenando et al. [6] in the same region of the Legal Amazon showed a delimitation of two well-defined climatic periods (dry and rainy). This fact influences concentration and dispersion of pollutants, given that pollution generated by biomass burning in the Amazon reaches its peak during the dry season (May-October). Results of PM_2.5 _modeled in the study region show that TS and PV areas presented much higher particulate matter values than the control area, this validating the choice of CH as a control in this study and corroborates with the toxicity evaluation study of Sisenando et al. [6] who also chose this same area as a control. With respect to meteorological variables, results presented show no significant difference between mean temperatures modeled among the areas. Higher humidity in Porto Velho in comparison to other points is justified by the fact that the area is part of the Amazon biome and consequently suffers greater influence from humidity emanating from the Amazon rainforest [39,40].

Baseline MN frequency obtained in CH (control area) is in agreement with values observed by Holland et al. [17] in the review article of the Human Micronucleus Project on Exfoliated Buccal Cells (HUMN_XL_) regarding the baseline observed in articles that evaluated environmental pollutants. Comparison between areas exposed and not exposed to pollution showed that children exposed to pollution have a higher frequency of micronuclei. These results corroborate studies by Maimulov et al. [28] in Russia, Lahiri et al. [30] India, Huen et al. [24] and Chen et al. [41] in the United States that evaluated the influence of air pollution in formation of micronuclei in children. The highest MN‰ observed in TS in relation to PV although not significant confirms the results of modeled particulate material on that TS was the area most exposed in both periods analyzed. This difference in particulate matter concentration observed in TS may be associated with burning of sugar cane in the area. Sisenando et al. [6] and Alves et al. [9] showed the genotoxic ability of pollutants generated by burning sugar cane in the TS region using the bioindicator *Tradescantia pallida in situ *and *ex situ *experiments. Alves et al. [9] also detected the presence of PAHs with high mutagenic potential (e.g.: benzo[e]pyrene) and n-alkanes in the sampled through Teflon filters in the troposphere of Tangará da Serra during the dry season.

In Brazil, consumption of vegetables, fruit and animal protein is associated with cultural values, and consumption of vitamin supplementation is expensive and restricted to individuals with greater buying power. Bonassi et al. [42] in a review article of the HUMN_XL _project shows that factors related to lifestyle and diet may influence frequency of micronuclei. In this study, the factor of alcohol and tobacco consumption was minimized at time of collection and the cultural factor was minimized by choosing areas (test and control) with similar cultural values. Although in the study region there is a clear economic difference between users of public and private schools, this fact did not provide significant differences between the two groups.

More recent publication HUMN_XL _project showed that people who eat fish regularly have a lower baseline MN frequency compared to non-consumers of fish [42].

When comparing household per capita consumption of fish between the two test areas, we found that fish consumption in PV was 2.31 times higher than in TS [43]. This may help to explain the low frequency of micronuclei observed in PV. Regarding gender, the results no showed significant difference between males and females. This result corroborates the review articles of Neri et al. [16] and Bonassi et al. [42] and did not corroborate the study of Maimulov et al. [28] which showed that female children were more sensitive to environmental pollution in St. Petersburg. In relation to age, the results showed a slight yet not significant increase in MN frequency in all areas. This observation is in accordance with Bonassi et al. [42] which showed that frequency of micronuclei constantly increases with age. In general, significant differences were observed when comparing data obtained in sites TS and PV with those from CH in all parameters, except between MN‰ in children under 07 years old from PV that was not significant in comparison to the control (*p > 0.05*). This fact can be explained by the small sample size in this age group.

A fact that could be considered a limitation of this study was the small number of children who participate in the study in the public school of Chapada dos Guimarães and the inability to conduct the study in the private school of Porto Velho. However, this study is marked by the pioneering character in the assessment of genotoxicity in an area of difficult access and with few environmental monitoring stations.

## Conclusions

In conclusion, the present study showed that in situ biomonitoring using a sensitive and low cost assay (buccal MN-assay) may be an important tool for monitoring air quality in the remote regions or which is not possible to have an air quality monitoring station. Considering that pollution generated by biomass burning is a complex mixture, it is difficult to attribute the increase in MN‰ observed in our study to any specific toxic element within in the particulate matters. However, the contribution of the present study lies the fact that we found significant evidence that increased exposure to PM_2.5 _generates an increased micronuclei frequency in oral epithelial cells from schoolchildren.

## Abbreviations

PM: Particulate Matter; PAHs: Polycyclic Aromatic Hydrocarbons; DNA: Deoxyribonucleic Acid; MCN-assay: Micronucleus Assay; MN: Micronuclei; TS: Tangará da Serra; PV: Porto Velho; CH: Chapada dos Guimarães; PM_2.5_: Fine Particulate Matter; CATT-BRAMS: Coupled Aerosol and Tracer Transport Model of the Brazilian Regional Atmospheric Modeling System; SISAM: Environmental Information Integrated to Environmental Health System; SPSS: Statistical Package for the Social Sciences; ANOVA: Analysis of Variance; MN‰: Mean Micronucleus Frequency; HUMN_XL_: Human Micronucleus Project on Exfoliated Buccal Cells; buccal MCN-assay: Buccal Micronucleus Assay.

## Competing interests

The authors declare that they have no competing interests.

## Authors' contributions

HAS participated in study design, conducted analyses, and drafted the manuscript; SRBM participated in study design, guided genotoxicity analyses, and reviewed manuscript drafts; PA was involved in revision and preparation of the manuscript for publication; PHNS was involved in revision and preparation of the manuscript for publication; SSH conceived the study, participated in its design and coordination and helped draft the manuscript. All authors read and approved the final manuscript.

## Pre-publication history

The pre-publication history for this paper can be accessed here:

http://www.biomedcentral.com/1472-6831/12/6/prepub
